# Platelets and the Cybernetic Regulation of Ischemic Inflammatory Responses through PNC Formation Regulated by Extracellular Nucleotide Metabolism and Signaling

**DOI:** 10.3390/cells11193009

**Published:** 2022-09-27

**Authors:** Tiago F. Granja, David Köhler, Veronika Leiss, Claudia Eggstein, Bernd Nürnberg, Peter Rosenberger, Sandra Beer-Hammer

**Affiliations:** 1 Lusófona’s Research Center for Biosciences & Health Technologies, CBIOS–Universidade, Campo Grande 376, 1749-024 Lisboa, Portugal; 2 Department of Anesthesiology and Intensive Care Medicine, Tübingen University Hospital, Wilhelmstrasse 56, D-72074 Tübingen, Germany; 3 Department of Pharmacology and Experimental Therapy and Toxicology and Interfaculty Center of Pharmacogenomics and Drug Research (ICePhA), Tübingen University Hospital, Wilhelmstrasse 56, D-72074 Tübingen, Germany

**Keywords:** platelets, ischemia, ischemic preconditioning, ATP, ADP, adenosine, VASP phosphory-lation, ADORAs, P2Y_12_

## Abstract

Ischemic events are associated with severe inflammation and are here referred to as ischemic inflammatory response (IIR). Recent studies identified the formation of platelet–neutrophil complexes (PNC) as key players in IIR. We investigated the role of extracellular platelet nucleotide signaling in the context of IIR and defined a cybernetic circle, including description of feedback loops. Cybernetic circles seek to integrate different levels of information to understand how biological systems function. Our study specifies the components of the cybernetic system of platelets in IIR and describes the theoretical progression of IIR passing the cybernetic cycle with positive and negative feedback loops based on nucleotide-dependent signaling and functional regulation. The cybernetic components and feedback loops were explored by cytometry, immunohistological staining, functional blocking antibodies, and ADP/ATP measurements. Using several ex vivo and in vivo approaches we confirmed cybernetic parameters, such as controller, sensor, and effector (VASP phosphorylation, P2Y_12_, ADORAs and GPIIb/IIIa activity), as well as set points (ADP, adenosine) and interfering control and disturbance variables (ischemia). We demonstrate the impact of the regulated platelet–neutrophil complex (PNC) formation in blood and the resulting damage to the affected inflamed tissue. Taken together, extracellular nucleotide signaling, PNC formation, and tissue damage in IIR can be integrated in a controlled cybernetic circle of platelet function, as introduced through this study.

## 1. Introduction

A cybernetic circle is a refined version of a recursive process creating a steady state. Most, if not all, physiological and biochemical processes are integrated in cybernetic circles [1]. Physiological processes, such as body temperature, glucose, and water balance, but also blood circulation and blood pressure are strictly controlled systems that can all be described as cybernetic circuits. Not only physiological but also pathophysiological processes, such as inflammation, are under the control of cybernetic circles.

As per classical definition, inflammation is characterized by redness (rubor), warming (calor), swelling (tumor), pain (dolor), and loss of function (function laesa). Inflammation can already be in progress, even if the classical symptoms are not yet visible or present [2]. Various processes can lead to inflammatory responses and not all of them are intentional, e.g., inflammatory autoimmune reactions. Inflammation can be the result of a cascade of events triggered by ischemia and reperfusion [3]. Thus, in this study we use the term “ischemic inflammatory response” (IIR) to demarcate it from other inflammatory processes in autoimmune diseases, infections, transplant rejection, cancer, or inflammation induced by the use of extra-corporeal blood pumping devices [4]. Ischemic inflammation is characterized by an orderly sequence of events involving the ischemic organ, the vessels, the circulating blood, and the lymphoid organs [3]. IIR can be categorically executed through secretion of mediators (e.g., chemokines, cytokines, and small molecules, such as ATP), activation of complement system, and changes in the activation status of different cell types (platelets, endothelium, epithelium, granulocytes, monocytes, erythrocytes) [5,6,7,8,9,10,11]. Here, we focus on platelets, their (de)activation status, and their inflammatory interaction with innate immune cells, i.e., neutrophils.

Platelets are important regulators of blood homeostasis, which is well defined as a cybernetic system for processes, such as aggregation or integrity of the vascular system [5,12,13,14]. In recent years, it has become increasingly clear, that platelets are crucial players in inflammation [15,16,17,18]. Platelets tightly control the action of neutrophils via the formation of platelet–neutrophil complexes (PNC) in the blood and guide the PNCs to the ischemic tissue. Thereby platelets convert neutrophils to deleterious effectors transmigrating into the ischemic tissue [19,20,21,22,23,24,25,26]. Nucleotide signaling and metabolism are key regulators in the activation and deactivation of platelets [27]. It is known that upon stimulation, platelets secrete ATP and ADP, which elicit platelet aggregation [28]. In particular, ADP as activator of the platelet receptors P2Y_1_ and P2Y_12_ is crucial for platelet activation and aggregation [27]. Recently, we could demonstrate that the inhibition of the P2Y_12_ signaling by G⍺_i2_-deficiency in murine platelets reduces the size of heart and brain infarcts [29]. These data and the blockage of the P2Y_12_-receptor (e.g., clopidogrel, ticagrelor, etc.) in ischemic therapy suggest a deleterious effect of ADP in IIR [6,30].

A rapid breakdown of extracellular ADP is crucial to prevent further platelet activation and recruitment. This task is mainly accomplished by the endothelial ectoNTPDase1 (CD39), which is highly expressed on the endothelium, to tightly control platelet reactivity [31,32]. Interestingly, NTPDase1 is also expressed on platelets themselves at a low level and is most likely essential to keep the platelet microenvironment in balance to prevent uncontrolled platelet activation through the rapid breakdown of low quantity of ATP/ADP [32]. NTPDase2 (CD39L1) is mainly present on the basolateral surface of endothelium, adventitia of vessels, and microvascular pericytes. NTPDase2 is functionally responsible for building up an ADP pool, as it catalyzes the conversion of ATP to ADP very quickly but that of ADP to AMP slowly [33,34]. The ecto-5′-nucleotidase CD73 converts extracellular AMP to adenosine, which is known to be protective in inflammation and IIR [35,36,37,38].

Adenosine elicits its physiological responses by binding to and activating one or more of the four transmembrane adenosine receptors (ADORAs), denoted A1, A2A, A2B, and A3 [39]. By signaling via ADORA2A and ADORA2B on platelets, adenosine inhibits platelet aggregation by increasing intracellular cAMP levels [40,41]. Moreover, adenosine does not only impede platelet activation, but it is also a highly potent inhibitor of leukocyte activation, thereby exerting anti-thrombotic and anti-inflammatory effects [42,43].

**Figure 1:**
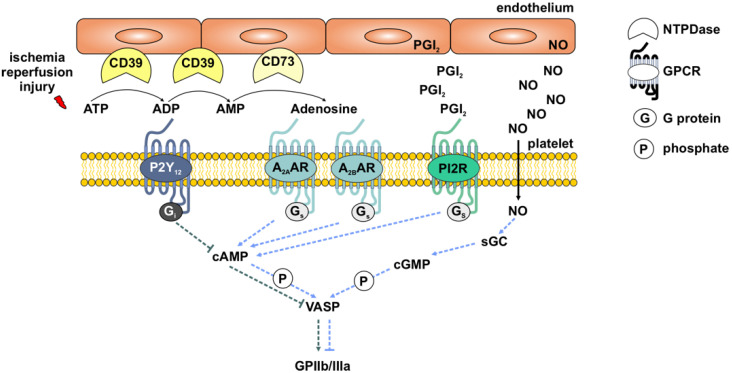
**Simplified scheme of physiological signaling pathways in platelets.** In platelets, G protein-coupled receptors (GPCR) act on the activation (P2Y_12_) and deactivation (A_2A_AR, A_2B_AR, PI2R) of Glycoprotein IIb/IIIa (GPIIb/IIIa) via the phosphorylation of vasodilator-stimulated phosphoprotein (VASP). NTPDases (CD39, CD73) expressed in the endothelium contribute to the breakdown of extracellular ATP and ADP to AMP and adenosine. Prostacycline (PGI_2_) and nitric oxide (NO) are released from the endothelium and support the deactivation of GPIIb/IIIa. ATP—adenosine triphosphate; ADP—adenosine diphosphate; AMP—adenosine monophosphate; cAMP—cyclic adenosine monophosphate; cGMP—cyclic guanosine monophosphate; sGC—soluble guanylyl cyclase; P2Y_12_—purinergic receptor; A_2A_AR—adenosine A_2A_ receptor; A_2B_AR—adenosine A_2B_ receptor.

In addition to this, platelet activation status is controlled by regulating the phosphorylation of vasodilator-stimulated phosphoprotein (VASP). VASP is known to control actin polymerization and filopodium formation [44,45]. The phosphorylation of VASP is driven by PKA and PKG, which are regulated by intracellular cAMP and cGMP levels, respectively [46,47]. In addition, the status of VASP-phosphorylation correlates with the activation of the integrin receptor complex GPIIb/IIIa [48,49]. Therapeutically, VASP phosphorylation status is determined as an indicator of platelet activation in patients to distinguish between P2Y_12_-responders and non-responders [49,50]. The activation status of GPIIb/IIIa can be measured with the specific antibodies PAC-1 and JON/A for human and murine platelets, respectively. It is well known that the activation of GPIIb/IIIa is essential for platelet aggregation, but its importance for the platelet–neutrophil complex (PNC) formation is still underestimated. These physiological pathways are summarized in Figure 1.

The aim of this study was to define a control circuit with the focus on the cell type platelets; their interplay with neutrophils in forming platelet–neutrophil complexes in the context of IIR before, during, and after an ischemic event. First, we specify all the components and correlations within this cybernetic cycle and then validate them with experimental approaches in murine models in vivo and in the human system ex vivo.

## 2. Materials and Methods

### 2.1. Ethic Statement

Following approval by the Institutional Review Board of Tübingen University Hospital (ethics committee), human blood samples were taken after written informed consent was gathered and neutrophils and platelets were isolated.

All animal experiments were performed in accordance with the German guidelines for use of living animals and were approved by the local authorities. For all animal experiments, wild type mice on a genetic C57BL/6 background were used and animals were selected with similar age, gender, and body weight.

### 2.2. Isolation of Human Platelets

Human venous blood was withdrawn in a 9 mL syringe with Na-Citrate (Monovette, Sarstedt, Nürnbrecht, Germany) and centrifuged without acceleration and break at 190× *g* for 20 min at room temperature (RT). The supernatant (PRP) was collected, and number of platelets determined.

### 2.3. ADP Release from Human Platelets

**Figure A1:**
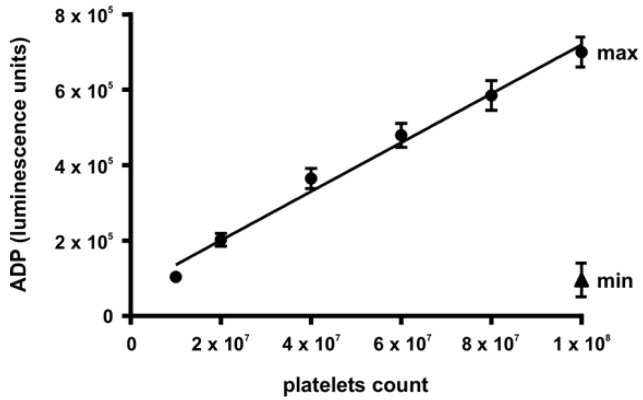
**Standard curve of ADP luminescence.** ADP release from 1 × 10^7^ to 1 × 10^8^ platelets was measured by enzymatic conversion of ADP to ATP measured in luminescence arbitrary units. Minimum ADP release taken from the supernatant of pelleted 1 × 10^8^ platelets (MIN), and maximum ADP release was assessed from the supernatant of 1 × 10^8^ platelets sonicated (MAX).

In 1 mL of HBSS resuspended platelets (1 × 10^8^ platelets/mL) the release of ADP was measured with an ADP/ATP ratio assay kit (ab65313; Abcam, Cambridge, UK). An ADP standard concentration curve (Chrono-Par^®^ from Chrono-Log^®^, Hamburg, Germany) was used to calculate the individual values (Figure A1) in platelet samples. Maximum ADP release was measured after sonication with 50% power for 10 s and minimum ADP release by centrifugation of platelets at 400× *g*, for 5 min at room temperature (RT). Shear stress was generated in platelet samples either with a 5 mL Potter-Elvehjem grinding chamber and Smooth Pestle with a chamber clearance of 100 to 150 µm to artificially simulate ex vivo microcirculatory vasculature (1×—one passage, 5×—five passages, 10×—ten passages), allowing the multiple passages of the pestle through the blood to mimic systolic thin viscosity observed under shear stress or with a pressure of 0.5 bar (equivalent to 370 mmHg, slightly above the hypertensive blood pressure), achieved with a 3 mL syringe with a stopper placed on an air pump for 10 s to mimic an ex vivo ischemic event [51].

### 2.4. Flow Cytometry of Human PRP

**Figure A2:**
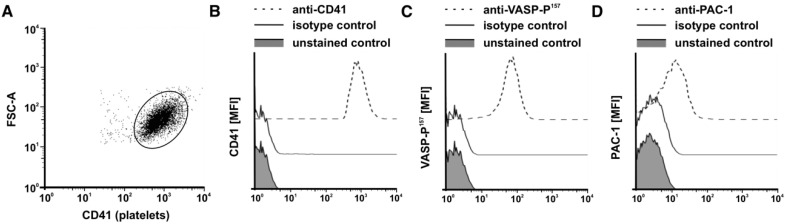
**Gating strategies for cytometry analysis shown in Figure 3 and Figure 4.** (**A**) Human platelets were labeled with CD41 antibodies to exclude cellular debris and microparticles. Staining control of (**B**) anti-human CD41 antibodies, (**C**) anti-human VASP-P^157^, and (**D**) anti-human PAC-1 antibodies, as compared to isotype control and unstained sample.

For the staining of surface markers (anti-human BV421-CD41 and APC-CD41/CD61 antibodies (clone PAC-1) (BioLegend, Koblenz, Germany)), platelet samples were incubated in 100 µL of staining solution for 20 min at RT and subsequently exposed to either 1 µg/mL PGE_1_, 12.5 µg/mL adenosine (Sigma-Aldrich Heidelberg, Germany), 25 µg/mL ADP (Chrono-Log^®^, Hamburg, Germany), or 5 µM Diethylamine NONOate diethylammonium salt (DEA NONOate; Cayman Chemical; Ann Arbor, USA) for 5 min, respectively, and after washing were resuspended in Cytofix™ (BD Biosciences, Heidelberg, Germany). For intracellular staining, samples were subsequently resuspended with Cytoperm™ (BD Biosciences, Heidelberg, Germany), centrifuged, and resuspended in Cytoperm™ solution to stain for 30 min supplemented with intracellular anti-human FITC-VASP-P^157^ or FITC-VASP-P^239^ antibodies (NanoTools, München, Germany). Prior to acquisition, platelet samples were washed twice and resuspended with perm/wash solution (BD Biosciences, Heidelberg, Germany). Fluorescence was evaluated on a FACS CantoII and data were analyzed using BD FACS DIVA Version 6.0 (BD Biosciences, Heidelberg, Germany). Respective staining controls are shown in Figure A2.

**Figure A3:**
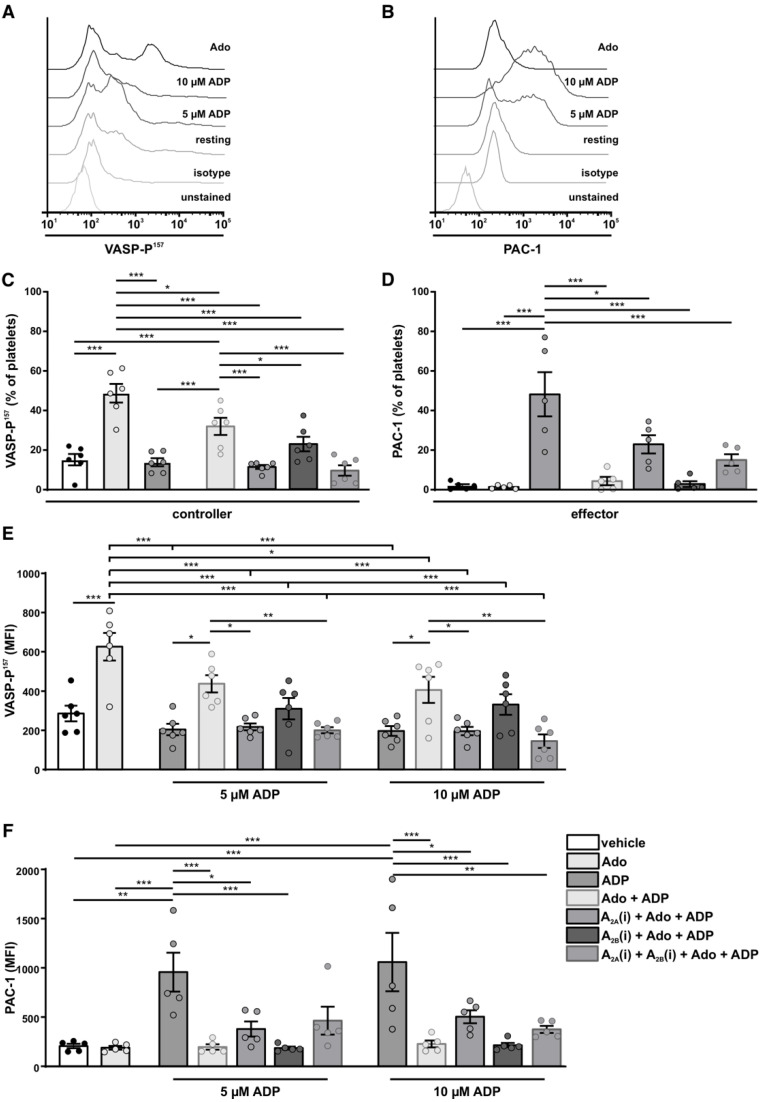
**Gating strategies and MFI for cytometry analysis shown in Figure 8.** Human platelets were gated with SSC and FSC. Histograms of (**A**,**C**) anti-human VASP-P^157^ and (**B**,**D**) anti-human PAC-1 antibody staining of unstained, isotype, resting, and adenosine or ADP-stimulated samples. (**E**) VASP phospohorylation at Serine^157^ is presented as mean fluorescence intensity (MFI). (**F**) Activation status of GPIIb/IIIa (PAC-1) is presented as mean fluorescence intensity (MFI). *n* = 5–6; one-way ANOVA with Tukey’s multiple comparisons test with * for *p* < 0.05; ** for *p* < 0.01; *** for *p* < 0.001.

To determine the impact of adenosine signaling via adenosine receptors A_2A_ and A_2B_ 1 × 10^6^ human platelets were stained with anti-human GPIIb/IIIa antibodies (PAC-1 clone, BioLegend, Koblenz, Germany) for 15 min at RT. Subsequently platelets were preincubated with adenosine A_2A_ and A_2B_ receptor inhibitors (10 µg/µL) (A_2A_(i)–ZM241385; A_2B_(i)–PSB1115; Tocris, Bristol, UK) or vehicle (0.05% DMSO) for 15 min, stimulated with 12.5 µg/mL adenosine for 30 min followed by 5 µM ADP stimulation for 20 min at RT. Platelets were fixed with 0.5% PFA prior acquisition. Respective staining controls are shown in Figure A3A.

For the analysis of VASP-phosphorylation platelets were stimulated, as described above; fixed with 1% PFA for 20 min and then permeabilized with 1× Permeabilization Buffer (ThermoFisher, Langenselbold, Germany); and stained with anti-human VASP-P^157^ antibodies (NanoTools, München, Germany) for 30 min. Platelets were washed with 1× permeabilization buffer and resuspended in PBS prior acquisition. Respective staining controls are shown in Figure A3B.

### 2.5. Flow Cytometry of Murine Whole Blood Samples

Adapted from a described methodology [52], for flow cytometry, 100 µL of murine whole blood were finally collected and stained with anti-mouse Ly6G (BioLegend, Koblenz, Germany), anti-mouse CD42b, and anti-mouse GPIIb/IIIa antibodies (JON/A clone, both from Emfret Analytics, Eibelstadt, Germany). Samples were incubated for 30 min at 37 °C and lysed with 1× BD lysis solution, washed with PBS (ThermoFisher, Langenselbold, Germany), resuspended in 1× Cytofix™ (BD Biosciences, Heidelberg, Germany) prior to acquisition on a FACS CantoII, and analyzed with BD FACS DIVA Version 6.0 (BD Biosciences, Heidelberg, Germany). Murine neutrophils in whole blood samples were gated for granularity (SSC-A) and Ly6G.

### 2.6. Hepatic and Myocardial Ischemia Reperfusion Model in Mice

A murine model of hepatic and myocardial ischemia reperfusion was employed using the previously described hanging weight system [53,54]. To detect PNCs in peripheral circulation, blood was withdrawn after one hour of ischemia followed by one minute of reperfusion and stained for flow cytometry analysis, as described previously [26]. To detect PNCs within the area at risk (AAR), hearts and livers were removed after one hour ischemia and one hour of reperfusion and processed for immunohistochemical staining, as described previously and below [26,29].

### 2.7. Neutrophils and Platelet Staining in Murine Hepatic and Myocardial Tissues

Immunohistological staining in paraffin-embedded/cryo sections of heart and liver was accomplished using Vectastain ABC Kit (Linaris, Wertheim, Germany). Avidin blocking (Vector; Burlingame, CA, USA) was used to avoid unspecific binding. After blocking, the tissue slices were incubated with the primary antibodies (rabbit anti-mouse CD41; Abcam, Cambridge, UK) for 16 h at 4 °C followed by incubation with biotinylated anti-rabbit immunoglobulin for 60 min. Subsequently, Vectastain ABC Reagent^®^ was applied for 30 min and the staining was detected by DAB substrate. For specific staining of neutrophils, the procedure was repeated using rat anti-mouse neutrophil antibodies (Ly-6B2 clone 7/4; AbD Serotec, Düsseldorf, Germany) and Histogreen as substrate (Linaris, Wertheim, Germany). For structural staining of the tissues, nuclear fast red (Linaris, Wertheim, Germany) was used.

### 2.8. Detection of Adenosine Receptors (ADORAs) in Human Platelets by Immunoblot Analysis

To detect expression of ADORA1, ADORA2A, ADORA2B, and ADORA3, human platelets were isolated and resuspended in RIPA buffer. Protein concentrations were measured by standard BCA method following the manufacturers’ instructions (Thermo Fisher Scientific, Hanover Park, IL, USA). Protein lysates (50 µg) were loaded on 10% SDS acrylamide gels and blotted onto nitrocellulose membranes, as described [55]. The antibodies detecting ADORA1 (sc-28995), ADORA2A (sc-13937), ADORA2B (sc-28996), and ADORA3 (sc-19388) were purchased from SantaCruz (Santa Cruz Biotechnology, Santa Cruz, CA, USA). β-Actin (Santa Cruz, CA, USA) served as loading control. A horseradish peroxidase (HRP)-conjugated anti-rabbit IgG antibody served for immunodetection (Santa Cruz Biotechnology, Inc., Santa Cruz, CA, USA). Immunoreactive bands were visualized by Westar ECL HRP substrate (Westar, Cyanagen, Italy).

### 2.9. Data Analysis

Data were checked for normal distribution. Statistical analyses were performed using one-way ANOVA with Tukey’s multiple comparisons test to determine group differences or unpaired Student’s t test where appropriate. This is indicated in the figure legends. A value of *p* < 0.05 was considered to be statistically significant.

## 3. Results

### 3.1. Layout of the Cybernetic System/Control Circuit “Platelets in IIR”

**Figure 2:**
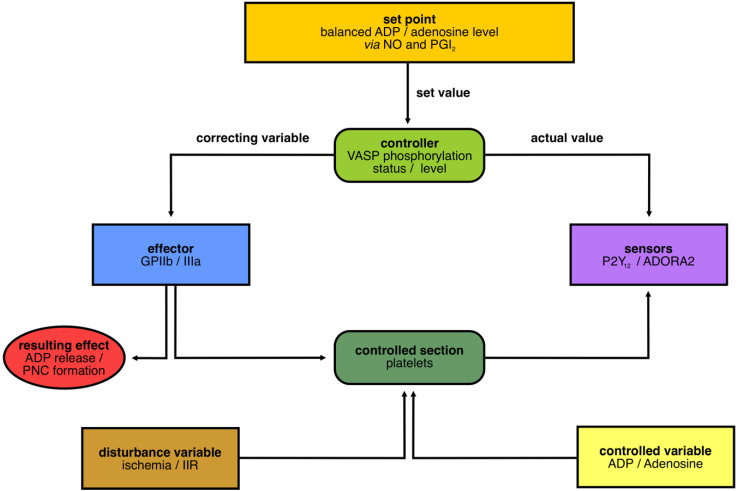
**Cybernetic cycle/control circuit in ischemic inflammatory response (IIR) focused on platelets.** To describe the role of platelets and their nucleotide metabolism for PNC formation, we first defined a cybernetic system with a balanced ADP/adenosine level as the set point, with the controller VASP, the effector GPIIb/IIIa, with the platelets as controlled section and P2Y_12_ and ADORA2 as main sensors. The set point is kept in balance via NO and PGI_2_. The phosphorylation status of the controller VASP regulates the (de)activation status of GPIIb/IIIa. This results in ADP release and PNC formation. Furthermore, disturbance variables, such as ischemia or ischemic inflammatory responses (IIR), act on the controlled section platelets and are counterregulated by the controlled variables ADP and Adenosine.

To determine our control circuit for “platelets in IIR”, we first defined platelets as the “controlled section” that have to be kept in homeostasis (Figure 2). Second, the “set point” was defined as the balanced ratio of extracellular ADP and adenosine (Ado), which is mainly controlled by nitric oxide (NO) and Prostacyclin (PGI_2_) [56] (Figure 2). “Disturbances”, such as ischemia or IIR, bring the system out of balance, and the components of a cybernetic system must react to bring the system back in homeostasis. In case of a “disturbance”, the “variable” ADP is released and detected by the “sensor” P2Y_12_. Subsequently, downstream signals in the platelets cause a change in the phosphorylation status of the “controller” VASP (dephosphorylation), which, in turn, causes a switch to the active state of the “effector”, the integrin complex GPIIb/IIIa. This activation is required for the binding of fibrinogen and for the subsequent formation of platelet–neutrophil complexes (PNCs), which we defined as our “resulting effect”, and subsequent processes, such as, degranulation were not considered.

The main “controlled variable” here is adenosine keeping the system in an inactive condition.

### 3.2. Initial Disturbance and the Positive Feedback Loop in the Nucleotide-Controlled Cybernetic System

**Figure 3:**
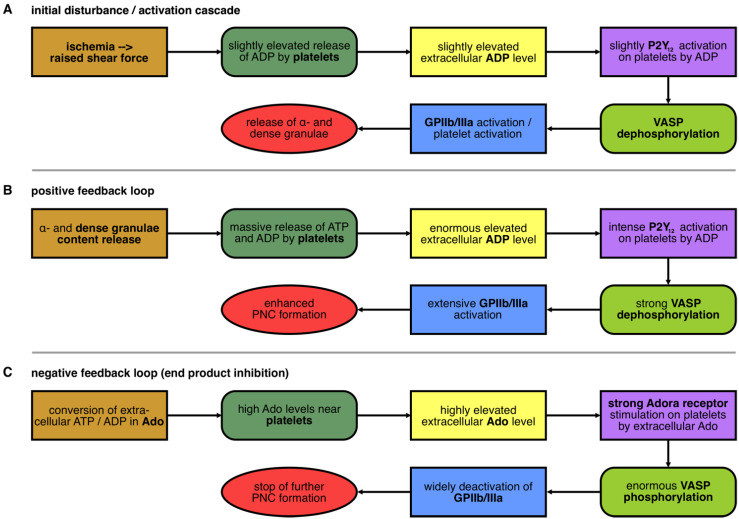
**Theoretical description of initial disturbance and feedback loops.** Different events are considered under the aspect of the described cybernetic circle. The different defined components of the cybernetic circle are illustrated in form and color, as in Figure 1, and describe the following three physiological processes. (**A**) Initial disturbance by ischemia induces shear force and subsequently results in release of α- and dense granule. (**B**) This starts the positive feedback mechanism. (**C**) The metabolite adenosine (Ado; end product of nucleotide metabolism) is initiating the negative feedback loop.

On the one hand, the initial activation of platelets by ADP (Figure 3A) is followed by an additional release of ADP, resulting in a positive feedback loop (Figure 2B) and massive PNC formation in the blood. On the other hand, the breakdown of extracellular ADP to Adenosine (Ado) serves as a negative feedback loop (Figure 3C). Both feedback mechanisms are elaborated in detail in the following sections. In IIR, the controlled section “platelets” are an autonomous working unit carrying all required ingredients. Soluble mediators needed for autocrine activation are stored in vesicles, the α- and dense granules, and can be released into the extracellular environment in the blood. The initial activation of platelets by e.g., an ischemic event, leads to the secretion of ADP inducing the activation of the P2Y_12_ receptor, VASP dephosphorylation, GPIIb/IIIa activation, and, finally, to an enhanced release of ADP from platelet granules (Figure 3A). This massive release of ATP and ADP is leading to an enormous amplification (positive feedback mechanism) via the sensor P2Y_12_. Inside the platelet, the controller VASP is strongly dephosphorylated and the effector GPIIb/IIIa is switched into an active state. This exacerbates the immediate formation of PNCs by binding fibrinogen and bridging the platelets to neutrophils (Figure 3B).

### 3.3. The Negative Feedback Loop in the Nucleotide-Controlled Cybernetic System

Whereas ADP is an important agonist at the P2Y_12_ receptor for platelet activation, the metabolite adenosine (Ado) serves as the trigger to bring the controlled section platelets back into a steady state equilibrium. Ado is the end product of the conversion of extracellular ADP by the ectoNTPDase1 (CD39) and ecto-5′-nucleotidase (CD73). Ado binds to the respective ADORA receptors A2A and A2B on platelets, thus increasing intracellular cAMP levels and leading to phosphorylation of the controller VASP. Consequently, the effector GPIIb/IIIa that actively orchestrates platelet aggregation and inflammatory association with immune cells (monocytes, neutrophils) is inactivated. This prevents further binding of the bridging molecule fibrinogen to GPIIb/IIIa, thereby returning the comprehensive activation status of platelets to basal homeostasis (Figure 3C).

Taken together, the two feedback mechanisms are controlled by two different types of sensors, the P2Y_12_ and the ADORA receptors A2A and A2B. Therefore, the levels of the “controlled variables” ADP and Ado are the major natural substances controlling the “controlled section” platelets and, therefore, consequently, their interaction with neutrophils.

In order to verify our theoretical model, the individual cybernetic components were experimentally addressed and are described in the following paragraphs.

### 3.4. Analysis of Set Point Components for Homeostasis

In the described cybernetic circle, PGI_2_ and NO are defined as two major set points inhibiting platelet aggregation and activation. Whereas the PGI_2_ receptor (PTGIR = prostaglandin I_2_ receptor) is expressed on the platelet surface, NO signals via intracellular soluble guanylyl cyclases [57,58,59]. However, both set points induce VASP phosphorylation mediated through cAMP/cGMP-induced activation of PKA/PKG [60,61].

**Figure 4:**
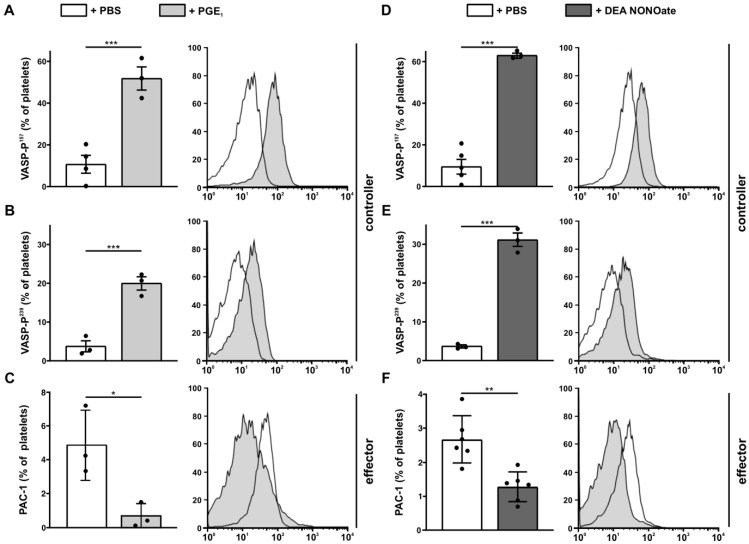
**Analysis of the set points.** Human platelets were treated with (**A**–**C**) either PGE_1_ (the more stable variant of PGI_2_) or (**D**–**F**) the NO donor DEA NONOate. Phosphorylation status at position (A+D) Ser^157^ and (B+E) Ser^239^ is depicted as percentage of platelets with respect to isotype controls (isotype controls and gating strategies are shown in Figure A2). (C+F) Activation status of GPIIb/IIIa was measured by PAC-1 binding and is presented as percentage of positively stained platelets with respect to isotype control (isotype controls, gating strategies, and MFIs are shown in Figure A2 and Figure A4). (**A**–**F**) One representative histogram is shown for each condition in the right panels (black outlined histogram: untreated, grey filled histogram: treated). *n* ≥ 3; unpaired Student’s *t*-test with * for *p* < 0.05; ** for *p* < 0.01; *** for *p* < 0.001.

**Figure A4:**
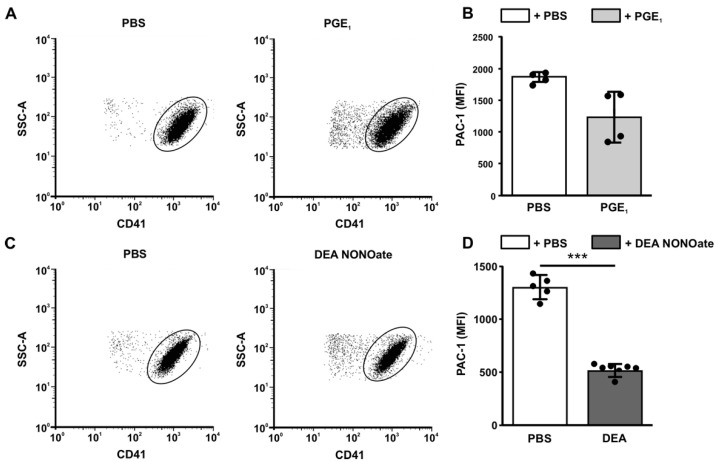
**Gating strategies and MFI for cytometry analysis shown in Figure 3.** Human platelets were treated with (**A**,**B**) either PGE_1_ or (**C**,**D**) DEA NONOate. (**A**,**C**) Human platelets were gated according to SSC and CD41. (**B**,**D**) Activation status of GPIIb/IIIa (PAC-1) is presented as mean fluorescence intensity (MFI). *n* ≥ 4; unpaired Student’s *t*-test with *** for *p* < 0.001.

Since we defined the phosphorylation of VASP as a controller, we first investigated whether changing the levels of the two set points would alter the phosphorylation status of VASP. Therefore, we stimulated human platelets with PGE_1_, a more stable form of PGI_2_, (Figure 4A–C) or with the NO donor DEA NONOate (Figure 4D–F) and analyzed both VASP-phosphorylation and the activation of the effector GPIIb/IIIa by PAC-1 antibody, respectively. As expected, treatment with PGE_1_ (Figure 4A,B) and DEA NONOate (Figure 4D,E) induced the phosphorylation of VASP on both serine-sites, Ser-157 and Ser-239. Subsequently, this treatment resulted in reduced GPIIb/IIIa activation, as measured by relative percentage of PAC-1 positive platelets (Figure 4C,F) and MFI of PAC-1 binding response (Figure A4).

Thus, PGI_2_ (as analyzed by PGE_1_) and NO showed typical features of a set point, which can keep the system in homeostasis by balancing ADP and Ado levels. On the contrary, during massive disturbances, such as ischemia, additional regulation by positive and negative feedback loops take place.

### 3.5. Analysis of the Initial Disturbance Induced by Ischemia

**Figure 5:**
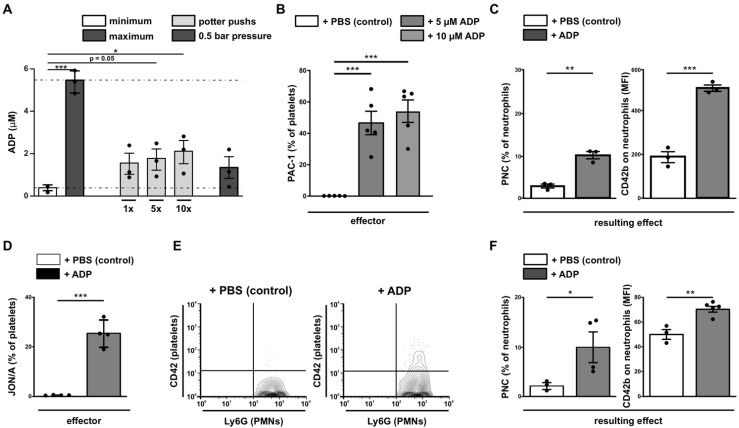
**Analysis of initial disturbance and positive feedback loop.** (**A**) Measurement of ADP release from human platelets induced by shear stress. Minimum ADP release was induced by centrifugation of platelets with 400× *g*, 5 min at RT; maximum ADP release was measured after sonication with 50% power for 10 s. Shear stress induction was generated with a Potter-Elvehjem (1×—one passage, 5×—five passages, 10×—ten passages) or with a pressure of 0.5 bar. (**B**) Human platelets were incubated with ADP (5 and 10 µM) (grey bars) and activation of GPIIb/IIIa was measured with PAC-1-binding and is depicted as percentage of PAC-1 positive platelets with respect to isotype control (one representative histogram is shown in Figure A5A). (**C**) Human neutrophils in whole blood samples were gated for size (FSC) and granularity (SSC-A). ADP-induced platelet–neutrophil complex (PNC) formation in human whole blood depicted as percentage of CD42b positive neutrophils (left panel) and by the platelet specific marker CD42b mean fluorescence intensity (MFI) measured on the surface of neutrophils. (**D**) Murine whole blood was analyzed accordingly using murine GPIIb/IIIa activity-specific JON/A antibodies and is depicted as percentage of platelets. (**E**) Representative contour plots for unstimulated and ADP-stimulated measurements. (**F**) Murine neutrophils in whole blood samples were gated for granularity (SSC-A) and Ly6G. ADP-induced platelet–neutrophil complex (PNC) formation in murine whole blood depicted as percentage of CD42b positive neutrophils (left panel) and by the platelet specific marker CD42b mean fluorescence intensity measured on the surface of neutrophils (right panel; corresponding histograms are depicted in Figure A5B–F). *n* ≥ 3; one-way ANOVA with Tukey’s multiple comparisons test for (**A**,**B**) and unpaired Student’s *t*-test for (**C**–**F**) with * for *p* < 0.05; ** for *p* < 0.01; *** for *p* < 0.001.

ADP is supposed to be the first natural activator for platelets during ischemia, induced by shear stress [62]. To verify this hypothesis, we have induced shear stress either by Potter-Elvehjem or by syringe pressure and were able to measure ADP release under both conditions (Figure 5A). This initial platelet activation by shear stress and the subsequent low release of ADP triggers the positive feedback loop, amplifying platelet activation.

### 3.6. Analysis of the Positive Feedback Loop

To mimic this positive feedback loop we stimulated human platelets with ADP and measured the activation status of the effector GPIIb/IIIa with the specific antibody PAC-1 As expected, treatment with ADP resulted in increased PAC-1 binding (Figure 5B). Before studying IRI in mice, we verified that GPIIb/IIIa activation (detected by JON/A antibodies) is also measurable in murine blood. Therefore, we analyzed ADP-stimulated murine whole blood using specific JON/A antibodies. Correspondingly, stimulation with ADP led to highly increased JON/A binding (Figure 5D). PNC formation was quantified as a resulting effect of the activation. In line with the induced activation of platelets, high numbers of PNCs were found in human and murine whole blood and are depicted either as a percentage of CD42b positive neutrophils (Figure 5C,E,F, left) or extent of CD42b expression (MFI) detected on neutrophils (Figure 5C,E,F, right).

### 3.7. Ischemia-Induced Changes in Platelet Activation Results in PNC Formation

It is well described that ischemia triggers the activation and transmigration of neutrophils into the infarcted area and the neutrophils are well known to be crucial for provoking reperfusion injury [63,64,65]. Meanwhile it is well established that the platelets guide the neutrophils, and the PNCs play a major role for adhesion, diapedesis, migration, and activation of neutrophils [66,67,68,69].

**Figure 6:**
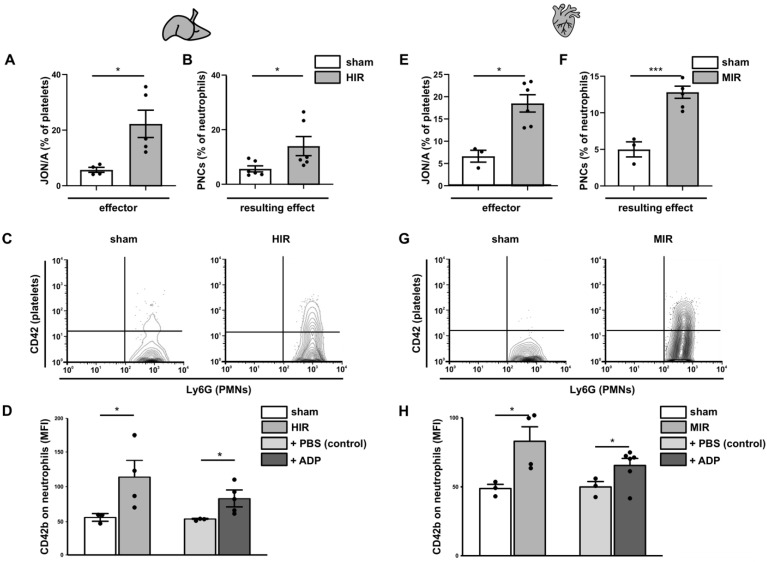
**Ischemia and PNC formation.** Hepatic (HIR; **A**–**D**) and myocardial (MIR; **E**–**H**) ischemia was induced followed by reperfusion in mice. (**A**,**E**) Platelet activation was assessed in whole blood by staining with JON/A antibodies and is depicted as percentage of platelets. (**B**,**F**) PNC formation was depicted as percentage of CD42b positive neutrophils. (**C**,**G**) Representative contour plots of (**B**) and (**F**), respectively. (**D**,**H**) Additionally, mean fluorescence (MFI) of CD42b positive platelets measured on the surface of neutrophils from sham (white bars) and IR mice (black bars) is depicted. Blood from sham mice was incubated ex vivo with PBS (grey) or ADP (dark grey). *n* = 3–5 mice; unpaired Student’s *t*-test with * for *p* < 0.05; *** for *p* < 0.001.

Therefore, we analyzed platelet activation and PNC formation in two different ischemia reperfusion (IR) models, the hepatic (HIR) (Figure 6 left panel), and the myocardial (MIR) (Figure 6 right panel).

First, we determined the activation state of the GPIIb/IIIa receptor using JON/A- antibody as an indicator for circulatory platelet activation (Figure 6A,E). As expected, both ischemia reperfusion models induced high levels of activated GPIIb/IIIa, in comparison to sham surgery. GPIIb/IIIa activation facilitates fibrinogen binding and bridging to macrophage-1 antigen (MAC-1) on neutrophils, resulting in PNC formation (Figure 6B,C,F,G). Next, blood from sham-operated and HIR- and MIR-treated mice was analyzed for PNC formation (Figure 6D,H). Interestingly, the percentage of circulating PNCs in the blood of HIR- and MIR-treated mice was significantly higher, compared to sham-operated blood samples and slightly higher, compared to blood samples of sham-operated mice incubated in vitro with ADP (Figure 6D,H).

**Figure 7:**
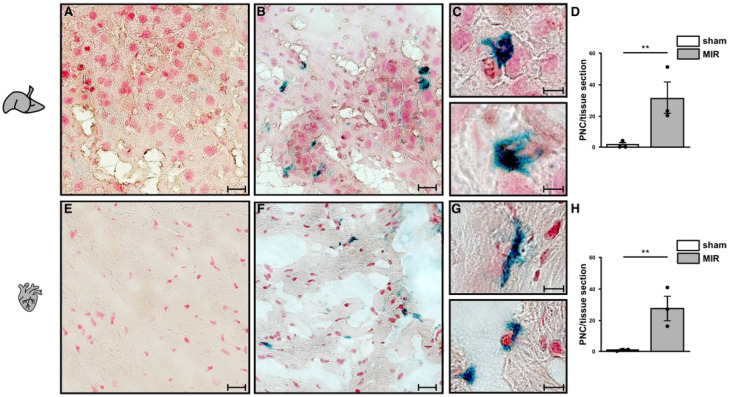
**Immunohistological analysis of PNCs within the infarcted tissue.** Representative histological images of liver (**A**–**C**) and heart tissue (**E**–**G**) without ischemia (**A**,**E**) and after IR (**B**,**F**). Scale bars 10 µm. PNCs were stained for neutrophils (anti-Ly-6B2; blue) and platelets (anti-CD41; black). Selected (**C**) liver and (**G**) heart sections magnification (1000×) showing neutrophils (blue) co-stained with platelets (black). Structural staining of organ cells was achieved by nuclear fast red. Control negative and IgG staining are shown in Figure A6 (liver) and Figure A7 (heart). Statistical analysis of PNCs in (**D**) liver and (**H**) heart sections. *n* = 3 mice; unpaired Student’s *t*-test with ** for *p* < 0.01.

In addition, the co-staining of platelets (black) and neutrophils (blue) in the affected tissue sections from HIR and MIR models revealed neutrophil infiltration to the infarcted area in the form of PNCs in tissue sections only from HIR- (Figure 7B,C) and MIR-subjected mice (Figure 7E,F) but not from sham operated mice. Importantly, we never observed any neutrophils without adherent platelets in the infarcted area, highlighting the crucial role of platelets and PNC formation for the progress of reperfusion injury after ischemic events.

### 3.8. Analysis of the Negative Feedback Loop

**Figure 8:**
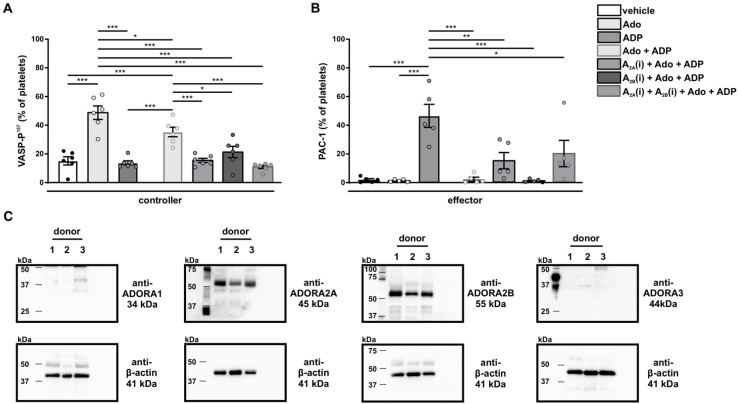
**Analysis of the negative feedback loop.** (**A**) Percentage of VASP-P^157^ phosphorylation and (**B**) GPIIb/IIIa activity (PAC-1) in human platelets after treatment with 12.5 µg/mL Ado and 5 µM ADP and in the presence of ADORA_2A_ and ADORA_2B_ inhibitors (10 µg/µL), as indicated. Additionally, stimulation of platelets with 10 µM ADP is shown in Figure A3. *n* ≥ 3; one-way ANOVA with Tukey’s multiple comparisons test with * for *p* < 0.05; ** for *p* < 0.01; *** for *p* < 0.001. (**C**) Immunoblot analysis of ADORA subtypes in human platelets of three donors. One representative image for each ADORA receptor (upper panel) and the corresponding β-actin control (lower panel) out of three experiments is shown.

During and after ischemic incidents the controlled section ‘platelet’ is massively dysregulated, and the set points (PGI_2_ and NO) are not sufficient to re-establish the system balance. Thus, Ado, derived from the breakdown of ADP, is additionally needed to restore homeostasis. To prove this, human platelets were treated with Ado and subsequently VASP-phosphorylation was measured. In line with our hypothesis, increased VASP phosphorylation at Serine157 was observed upon Ado treatment, whereas ADP treatment had no impact on VASP phosphorylation (Figure 8A left panel). Accordingly, human platelets treated with Ado showed a significant deactivation of the effector GPIIb/IIIa, whereas the effector was significantly activated after ADP stimulation. (Figure 8B left panel). Signal transduction of Ado is mediated through the ADORA receptors. Two of these four receptors, namely ADORA1 and ADORA3, are known to downregulate intracellular cAMP levels upon Ado stimulation [70,71]; however, they are not involved in the described pathway of VASP-regulation [72,73,74]. In addition, the proteome database reports no expression of ADORA1 and ADORA3 in platelets (https://plateletweb.bioapps.biozentrum.uni-wuerzburg.de/plateletweb.php, accessed on 12 June 2022). In line with this, ADORA 1 and 3 expression was hardly detectable in human platelets. Therefore, ADORA 1 and 3 receptor function was not further analyzed. In contrast, strong expression of ADORA_2A_ and ADORA_2B_ was found (Figure 8C). This, together with the fact that activation of ADORA2 leads to an increase in intracellular cAMP levels (Figure 1), suggests that VASP phosphorylation is mainly regulated via ADORA_2A_ and ADORA_2B_ in platelets [75]. To validate that adenosine signals via ADORA2 receptors, platelets were pretreated with adenosine and subsequently stimulated with ADP in the absence or presence of A_2A_- and/or A_2B_-specific inhibitors (Figure 8A,B). On the one hand, blocking specifically ADORA_2A_ or ADORA_2B_ reduced Ado-induced phosphorylation of VASP to resting and ADP conditions (Figure 8A right panel). On the other hand, GPIIb/IIIa activation was induced by ADP treatment and the protective effect of adenosine was diminished in the presence of ADORA_2A_ inhibitors (Figure 8B).

This emphasizes the role of ADORA2 receptors in the interplay of activation and deactivation of platelets. Therefore, this signaling cascade can be defined as the negative feedback loop.

## 4. Discussion

The physiological functions of platelets to keep the integrity of the vasculature and homeostasis have been well described for several decades, whereas the inflammatory role of platelets has long been neglected. In recent years, the role of platelets in inflammatory processes has been intensively investigated [76,77,78]. In this context, the function of platelets in different diseases caused mainly by inflammatory stimuli, such as sepsis, lung injury, and colitis, were analyzed [79,80,81,82]. In addition to this, the role of platelets was also examined in ischemia, focusing on thrombus formation, leading to vessel occlusion and, subsequently, infarction. Based on the role of platelets and PNCs in influencing the outcome in ischemia-reperfusion injury [23,24,25,26,83,84,85], we propose that platelets are crucial in controlling and directing neutrophils to the infarcted area, thus enabling the destruction of the tissue by neutrophils during the acute inflammatory phase following reperfusion. Thus, platelets are regulating the ischemic inflammatory responses (IIR) by forming platelet–neutrophil complexes (PNCs) and guiding the neutrophils into the ischemic tissue causing reperfusion injury [86]. The initial step in the formation of PNCs is the connection of P-Selectin to PSGL-1, which results in stable PNC formation, further aided by GPIIb/IIIa binding to MAC-1 [87,88,89] bridged by fibrinogen. Most of these aspects of activation and function of platelets are well known and extensively investigated.

Here, we consider the components of the control circuit from the perspective of extracellular nucleotide signaling in the context of ischemia and the resulting ischemia reperfusion injury with platelets as the “controlled section“ of this cybernetic system.

Changes in the phosphorylation status of the “controller” VASP directly regulates the “effector” GPIIb/IIIa integrin receptor, with the latter mainly reflecting the overall activation status of the platelets. As shown here and by others, ADP activates platelets, resulting in a conformational change of the GPIIb/IIIa receptor to allow fibrinogen binding [90,91]. The receptors for extracellular nucleotides P2Y_12_ (ADP) and ADORA_2A_ and ADORA_2B_ (Ado) represent the “sensors” that alter the controller status appropriately in response to physiological demands. Agonist-induced desensitization should be considered for these GPCRs, which has to be examined in follow-up studies. In this control circuit, a balanced ADP/adenosine level (“set point”) is crucial, which is achieved by a constant release of NO and/or effected by PGI_2_. “Disturbance variables”, such as IIR, are misbalancing the homeostasis, resulting in massive ADP release, PNC formation, and ischemic reperfusion injury [92].

Our description of the cybernetic system allows us to explain already known correlations during ischemia, e.g., it is known that in mice, depletion of platelets prior to ischemia prevents reperfusion injury [23,93,94]. Our definition of the components within the control circuit emphasizes the importance of the cell type ‘platelet’ in controlling the role of neutrophils not only in inflammation but also in IIR [86]. In this context, the importance of the controller VASP was demonstrated in myocardial IRI showing the relevance of VASP phosphorylation for reducing IRI by stimulation with the set point parameter, PGE_1_ [23,24]. In addition, we could show that the controller VASP is crucial for the tissue protective effect in myocardial ischemic preconditioning (IP) [25]. The protective effect of IP was abrogated in VASP-deficient mice due to lack of VASP phosphorylation. We suggest that an optimal short ischemic timeframe in IP might trigger ATP/ADP release by platelets, which could be counteracted by an immediate reperfusion phase so that the platelets are deactivated by the action of their set points (PGI_2_ and NO) [65,95]. Another supporting effect is the initiated negative feedback loop, generating extracellular adenosine and, therefore, inactivating the platelets through the described ADORA_2A_ and ADORA_2B_ receptor signaling by phosphorylating VASP and, therefore, inactivating the effector GPIIb/IIIa. The optimal number of cycles is accomplished when all ATP/ADP stores in the dense granules of the platelets are exhausted and no further activation by the positive feedback loop is possible. According to this point of view, the known and described effect of remote IP is explicable, as empty granule stores in platelets will result in diminished PNC formation, adhesion, and transmigration, and, therefore, platelets will no longer able to direct neutrophils to the ischemic tissue. Based on these facts, we consider VASP as the major controller of platelet activation to regulate PNC formation.

It has to be acknowledged that volatile anesthetics, e.g., sevoflurane, induce VASP phosphorylation [42]. For sevoflurane and isoflurane, it was shown that they massively phosphorylate other actin-related proteins and regulate, therefore, the F-actin and G-actin ratio, resulting in dissolution of actin filaments in neuroblastoma cells [96]. Additionally, it was shown for isoflurane that it directly influences MIRI [97]. Therefore, anesthetics for animal ischemia reperfusion experiments have to be selected very carefully to avoid any pre-protection in the cybernetic model while using VASP phosphorylating agents. Certainly, there are VASP-independent protective mechanisms in ischemia/reperfusion, which are mainly important in adhesion, transmigration, and extravasation but less in PNC formation and/or platelet activation/deactivation.

In patients, the status of the controller VASP is used as a measurement of responsiveness to P2Y_12_ blockers, such as Clopidogrel. These drugs impair the activation of platelets and subsequent formation of PNCs and, therefore, protect from tissue injury.

We now suppose that the described circle can be adopted to a platelet-specific in vitro test system to develop and validate new therapeutic medications, targeting ischemia reperfusion injury, which can be applied in the late phase of ischemia or the early phase of reperfusion. With this approach, it is possible to test multiple drugs in parallel using human platelets and measuring VASP phosphorylation at Ser^157^ and Ser^239^ (controller) and the activation status of the GPIIb/IIIa (effector) by PAC-1 antibodies.

## Figures and Tables

**Figure A5:**
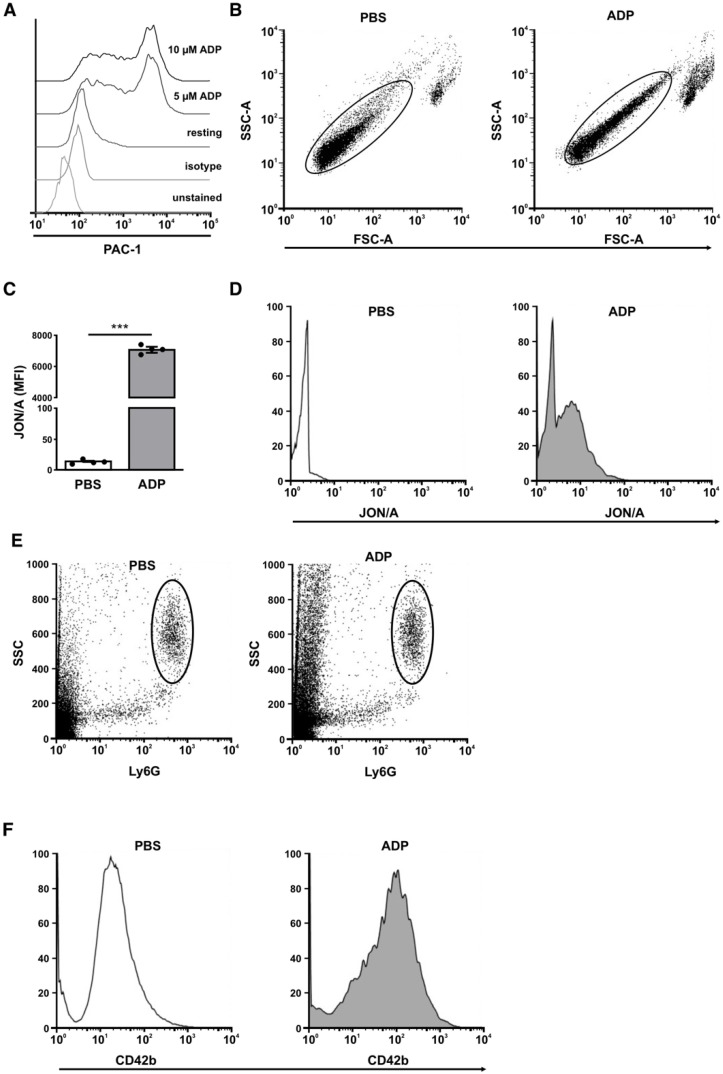
**Gating strategies and MFI for cytometry analysis shown in Figure 5.** (**A**–**C**) Human PRP or whole blood was treated with either PBS or ADP. (**D**–**F**) Murine PRP or whole blood was treated with either PBS or ADP. (**A**) Representative histograms showing GPIIb/IIIa activity using PAC-1 antibodies. (**B**) Platelets were gated according to SSC and FSC. (**C**) Mean fluorescence intensity (MFI) of JON/A. (**D**) Representative histograms showing GPIIb/IIIa activity using specific JON/A antibodies. (**E**) Neutrophils were gated according to SSC and Ly6G. (**F**) Representative histograms showing CD42b MFI of platelets on neutrophils. *n* ≥ 3; unpaired Student’s *t*-test with *** for *p* < 0.001.

**Figure A6:**
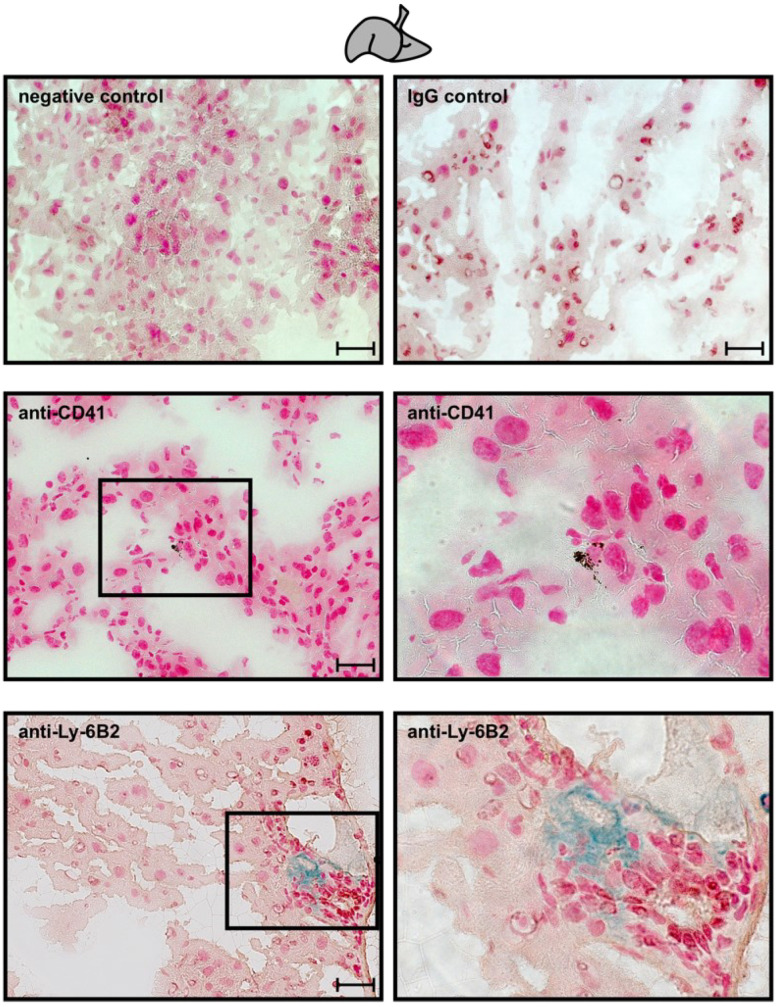
**Immunohistological staining of infarcted liver tissue**. Structural staining of organ cells was achieved by nuclear fast red. Immunohistological isotype and secondary antibody control staining (nuclear fast red stained without anti-CD41 or anti-Ly-6B2). Scale bars 10 µm. Representative single staining of platelets (anti-CD41) and neutrophils (anti-Ly-6B2), as indicated. **Right panels** are 2.5× magnifications of the corresponding **left panels**. Representative images of *n* = 3 mice are shown.

**Figure A7:**
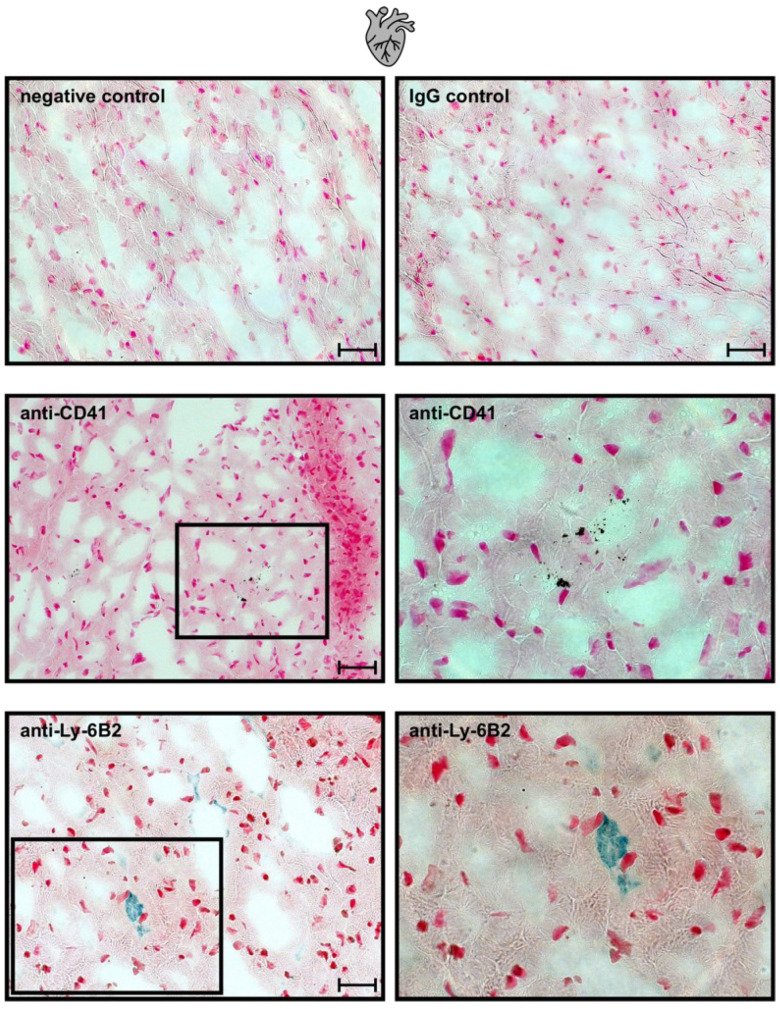
**Immunohistological staining of infarcted heart tissue**. Structural staining of organ cells was achieved by nuclear fast red. Immunohistological isotype and secondary antibody control staining (nuclear fast red stained without anti-CD41 or anti-Ly-6B2). Scale bars 10 µm. Representative single staining of platelets (anti-CD41) and neutrophils (anti-Ly-6B2), as indicated. **Right panels** are 2.5× magnifications of the corresponding **left panels**. Representative images of *n* = 3 mice are shown.

## Data Availability

The data presented in this study are available on request from the corresponding author upon reasonable request.

## References

[1] Wiener N. (1948). Cybernetics. Sci. Am..

[2] Punchard N.A., Whelan C.J., Adcock I. (2004). The Journal of Inflammation. J. Inflamm..

[3] Iadecola C., Anrather J. (2011). The immunology of stroke: From mechanisms to translation. Nat. Med..

[4] Granja T., Magunia H., Schüssel P., Fischer C., Prüfer T., Schibilsky D., Serna-Higuita L., Wendel H.P., Schlensak C., Häberle H. (2020). Left ventricular assist device implantation causes platelet dysfunction and proinflammatory platelet-neutrophil interaction. Platelets.

[5] Versteeg H.H., Magunia H., Schüssel P., Fischer C., Prüfer T., Schibilsky D., Serna-Higuita L., Wendel H.P., Schlensak C., Häberle H. (2013). New fundamentals in hemostasis. Physiol. Rev..

[6] Amison R., Page C., Pitchford S. (2012). Pharmacological modulation of the inflammatory actions of platelets. Handb. Exp. Pharmacol..

[7] Taub D.D. (1996). Chemokine-leukocyte interactions. The voodoo that they do so well. Cytokine Growth Factor Rev..

[8] Gopalakrishnan M., Saurabh S. (2014). Is red blood cell a mediator of remote ischaemic preconditioning?. Med. Hypotheses.

[9] Rodrigues S.F., Granger D.N. (2010). Role of blood cells in ischaemia-reperfusion induced endothelial barrier failure. Cardiovasc. Res..

[10] Frangogiannis N.G., Smith C.W., Entman M.L. (2002). The inflammatory response in myocardial infarction. Cardiovasc. Res..

[11] Gorsuch W.B., Chrysanthou E., Schwaeble W., Stahl G.L. (2012). The complement system in ischemia-reperfusion injuries. Immunobiology.

[12] Ruggeri Z.M., Mendolicchio G.L. (2007). Adhesion mechanisms in platelet function. Circ. Res..

[13] Jesty J., Beltrami E. (2005). Positive feedbacks of coagulation: Their role in threshold regulation. Arter. Thromb. Vasc. Biol..

[14] Kanthi Y., Hyman M.C., Liao H., Baek A.E., Visovatti S.H., Sutton N., Goonewardena S.N., Neral M.K., Jo H., Pinsky D.J. (2015). Flow-dependent expression of ectonucleotide tri(di)phosphohydrolase-1 and suppression of atherosclerosis. J. Clin. Investig..

[15] Rossaint J., Zarbock A. (2015). Platelets in leucocyte recruitment and function. Cardiovasc. Res..

[16] Semple J.W., Italiano J.E., Freedman J. (2011). Platelets and the immune continuum. Nat. Rev. Immunol..

[17] Herter J.M., Rossaint J., Zarbock A. (2014). Platelets in inflammation and immunity. J. Thromb. Haemost..

[18] Mezger M., Nording H., Sauter R., Graf T., Heim C., Von Bubnoff N., Ensminger S.M., Langer H.F. (2019). Platelets and Immune Responses During Thromboinflammation. Front. Immunol..

[19] Bazzoni G., Dejana E., del Maschio A. (1991). Platelet-neutrophil interactions. Possible relevance in the pathogenesis of thrombosis and inflammation. Haematologica.

[20] Mosesson M.W. (2005). Fibrinogen and fibrin structure and functions. J. Thromb. Haemost..

[21] Selak M.A. (1994). Neutrophil-platelet interactions in inflammation. Receptor.

[22] Peters M.J., Dixon G., Kotowicz K.T., Hatch D.J., Heyderman R.S., Klein N.J. (1999). Circulating platelet-neutrophil complexes represent a subpopulation of activated neutrophils primed for adhesion, phagocytosis and intracellular killing. Br. J. Haematol..

[23] Kohler D., Straub A., Weissmüller T., Faigle M., Bender S., Lehmann R., Wendel H.-P., Kurz J., Walter U., Zacharowski K. (2011). Phosphorylation of vasodilator-stimulated phosphoprotein prevents platelet-neutrophil complex formation and dampens myocardial ischemia-reperfusion injury. Circulation.

[24] Kohler D., Birk P., König K., Straub A., Eldh T., Morote-García J.C., Rosenberger P. (2011). Phosphorylation of vasodilator-stimulated phosphoprotein (VASP) dampens hepatic ischemia-reperfusion injury. PLoS ONE.

[25] Kohler D., Bibli S.-I., Klammer L.P., Roth J.M., Lehmann R., Fleming I., Granja T.F., Straub A., Benz P.M., Rosenberger P. (2018). Phosphorylation of vasodilator-stimulated phosphoprotein contributes to myocardial ischemic preconditioning. Basic Res. Cardiol..

[26] Kohler D., Granja T., Volz J., Koeppen M., Langer H.F., Hansmann G., Legchenko E., Geisler T., Bakchoul T., Eggstein C. (2020). Red blood cell-derived semaphorin 7A promotes thrombo-inflammation in myocardial ischemia-reperfusion injury through platelet GPIb. Nat. Commun..

[27] Idzko M., Ferrari D., Eltzschig H.K. (2014). Nucleotide signalling during inflammation. Nature.

[28] Gachet C. (2006). Regulation of platelet functions by P2 receptors. Annu. Rev. Pharmacol. Toxicol..

[29] Devanathan V., Hagedorn I., Köhler D., Pexa K., Cherpokova D., Kraft P., Singh M., Rosenberger P., Stoll G., Birnbaumer L. (2015). Platelet G_i_ protein G_αi2_ is an essential mediator of thrombo-inflammatory organ damage in mice. Proc. Natl. Acad. Sci. USA.

[30] Evangelista V., Manarini S., Dell’Elba G., Martelli N., Napoleone E., Di Santo A., Lorenzet P.S.R. (2005). Clopidogrel inhibits platelet-leukocyte adhesion and platelet-dependent leukocyte activation. Thromb. Haemost..

[31] Burnstock G. (2015). Blood cells: An historical account of the roles of purinergic signalling. Purinergic Signal..

[32] Koziak K., Sévigny J., Robson S.C., Siegel J.B., Kaczmarek E. (1999). Analysis of CD39/ATP diphosphohydrolase (ATPDase) expression in endothelial cells, platelets and leukocytes. Thromb. Haemost..

[33] Atkinson B., Dwyer K., Enjyoji K., Robson S.C. (2006). Ecto-nucleotidases of the CD39/NTPDase family modulate platelet activation and thrombus formation: Potential as therapeutic targets. Blood Cells Mol. Dis..

[34] Sevigny J., Sundberg C., Braun N., Guckelberger O., Csizmadia E., Qawi I., Imai M., Zimmermann H., Robson S.C. (2002). Differential catalytic properties and vascular topography of murine nucleoside triphosphate diphosphohydrolase 1 (NTPDase1) and NTPDase2 have implications for thromboregulation. Blood.

[35] Kohler D., Eckle T., Faigle M., Grenz A., Mittelbronn M., Laucher S., Hart M.L., Robson S.C., Müller C.E., Eltzschig H.K. (2007). CD39/Ectonucleoside Triphosphate Diphosphohydrolase 1 Provides Myocardial Protection During Cardiac Ischemia/Reperfusion Injury. Circulation.

[36] Eckle T., Köhler D., Lehmann R., El Kasmi K.C., Eltzschig H.K. (2008). Hypoxia-inducible factor-1 is central to cardioprotection: A new paradigm for ischemic preconditioning. Circulation.

[37] Morote-Garcia J.C., Köhler D., Roth J.M., Mirakaj V., Eldh T., Eltzschig H.K., Rosenberger P. (2013). Repression of the equilibrative nucleoside transporters dampens inflammatory lung injury. Am. J. Respir. Cell Mol. Biol..

[38] Kohler D., Streißenberger A., Morote-García J.C., Granja T., Schneider M., Straub A., Boison D., Rosenberger P. (2016). Inhibition of Adenosine Kinase Attenuates Acute Lung Injury. Crit. Care Med..

[39] Hasko G., Linden J., Cronstein B., Pacher P. (2008). Adenosine receptors: Therapeutic aspects for inflammatory and immune diseases. Nat. Rev. Drug Discov..

[40] Iyu D., Glenn J.R., White A.E., Fox S.C., Heptinstall S. (2011). Adenosine derived from ADP can contribute to inhibition of platelet aggregation in the presence of a P2Y_12_ antagonist. Arter. Thromb. Vasc. Biol..

[41] Yang D., Chen H., Koupenova M., Carroll S.H., Eliades A., Freedman J.E., Toselli P., Ravid K. (2010). A new role for the A2b adenosine receptor in regulating platelet function. J. Thromb. Haemost..

[42] Granja T.F., Köhler D., Schad J., Franz C.B.D.O., Konrad F., Hoch-Gutbrod M., Streißenberger A., Rosenberger P., Straub A. (2016). Adenosine Receptor Adora2b Plays a Mechanistic Role in the Protective Effect of the Volatile Anesthetic Sevoflurane during Liver Ischemia/Reperfusion. Anesthesiology.

[43] Gachet C., Hechler B. (2020). Platelet Purinergic Receptors in Thrombosis and Inflammation. Hamostaseologie.

[44] Ferron F., Rebowski G., Lee S.H., Dominguez R. (2007). Structural basis for the recruitment of profilin-actin complexes during filament elongation by Ena/VASP. Embo. J..

[45] Bachmann C., Fischer L., Walter U., Reinhard M. (1999). The EVH2 domain of the vasodilator-stimulated phosphoprotein mediates tetramerization, F-actin binding, and actin bundle formation. JBC.

[46] Halbrugge M., Friedrich C., Eigenthaler M., Schanzenbächer P., Walter U. (1990). Stoichiometric and reversible phosphorylation of a 46-kDa protein in human platelets in response to cGMP- and cAMP-elevating vasodilators. J. Biol. Chem..

[47] Walter U., Gambaryan S. (2009). cGMP and cGMP-dependent protein kinase in platelets and blood cells. Handb. Exp. Pharmacol..

[48] Frere C., Cuisset T., Quilici J., Camoin L., Carvajal J., Morange P.E., Lambert M., Juhan-Vague I., Bonnet J.-L., Alessi M.-C. (2007). ADP-induced platelet aggregation and platelet reactivity index VASP are good predictive markers for clinical outcomes in non-ST elevation acute coronary syndrome. Thromb. Haemost..

[49] Morel O., Ohlmann P., Jesel L., Desprez D., Grunebaum L., Bareiss P., Morel O. (2007). Impaired platelet responsiveness to clopidogrel identified by flow cytometric vasodilator-stimulated phosphoprotein (VASP) phosphorylation in patients with subacute stent thrombosis. Thromb. Haemost..

[50] Morel O., Bernhard N., Desprez D., Grunebaum L., Freyssinet J.M., Toti F., Bareiss P. (2008). Residual prothrombotic status in low responder patients to clopidogrel identified by Vasodilator-Stimulated Phosphoprotein Phosphorylation (VASP) analysis?. Thromb. Haemost..

[51] Cowan A.Q., Cho D.J., Rosenson R.S. (2012). Importance of blood rheology in the pathophysiology of atherothrombosis. Cardiovasc. Drugs Ther..

[52] Granja T., Schad J., Schüssel P., Fischer C., Häberle H., Rosenberger P., Straub A. (2015). Using six-colour flow cytometry to analyse the activation and interaction of platelets and leukocytes—A new assay suitable for bench and bedside conditions. Thromb. Res..

[53] Hart M.L., Much C., Köhler D., Schittenhelm J., Gorzolla I.C., Stahl G.L., Eltzschig H.K. (2008). Use of a hanging-weight system for liver ischemic preconditioning in mice. Am. J. Physiol. Gastrointest Liver Physiol..

[54] Eckle T., Grenz A., Köhler D., Redel A., Falk M., Rolauffs B., Osswald H., Kehl F., Eltzschig H.K. (2006). Systematic evaluation of a novel model for cardiac ischemic preconditioning in mice. Am. J. Physiol. Heart Circ. Physiol..

[55] Kohler D., Devanathan V., de Oliveira Franz C.B., Eldh T., Novakovic A., Roth J.M., Granja T., Birnbaumer L., Rosenberger P., Beer-Hammer S. (2014). G_αi2_- and G_αi3_-deficient mice display opposite severity of myocardial ischemia reperfusion injury. PLoS ONE.

[56] Broekman M.J., Eiroa A.M., Marcus A.J. (1991). Inhibition of human platelet reactivity by endothelium-derived relaxing factor from human umbilical vein endothelial cells in suspension: Blockade of aggregation and secretion by an aspirin-insensitive mechanism. Blood.

[57] Wang G.R., Zhu Y., Halushka P.V., Lincoln T.M., Mendelsohn M.E. (1998). Mechanism of platelet inhibition by nitric oxide: In vivo phosphorylation of thromboxane receptor by cyclic GMP-dependent protein kinase. Proc. Natl. Acad. Sci. USA.

[58] Riddell D.R., Owen J.S. (1999). Nitric oxide and platelet aggregation. Vitam. Horm..

[59] Gambaryan S., Subramanian H., Kehrer L., Mindukshev I., Sudnitsyna J., Reiss C., Rukoyatkina N., Friebe A., Sharina I., Martin E. (2016). Erythrocytes do not activate purified and platelet soluble guanylate cyclases even in conditions favourable for NO synthesis. Cell Commun. Signal..

[60] Shiravand Y., Walter U., Jurk K. (2021). Fine-Tuning of Platelet Responses by Serine/Threonine Protein Kinases and Phosphatases-Just the Beginning. Hamostaseologie.

[61] Makhoul S., Walter E., Pagel O., Walter U., Sickmann A., Gambaryan S., Smolenski A., Zahedi R.P., Jurk K. (2018). Effects of the NO/soluble guanylate cyclase/cGMP system on the functions of human platelets. Nitric. Oxide.

[62] Viisoreanu D., Gear A. (2007). Effect of physiologic shear stresses and calcium on agonist-induced platelet aggregation, secretion, and thromboxane A2 formation. Thromb. Res..

[63] Simpson P.J., Mickelson J., Fantone J.C., Gallagher K.P., Lucchesi B.R. (1988). Reduction of experimental canine myocardial infarct size with prostaglandin E1: Inhibition of neutrophil migration and activation. J. Pharmacol. Exp. Ther..

[64] Carini R., Albano E. (2003). Recent insights on the mechanisms of liver preconditioning. Gastroenterology.

[65] Jugdutt B.I. (2002). Nitric oxide and cardioprotection during ischemia-reperfusion. Heart Fail. Rev..

[66] Zarbock A., Singbartl K., Ley K. (2006). Complete reversal of acid-induced acute lung injury by blocking of platelet-neutrophil aggregation. J. Clin. Investig..

[67] Asaduzzaman M., Lavasani S., Rahman M., Zhang S., Braun O., Jeppsson B., Thorlacius H. (2009). Platelets support pulmonary recruitment of neutrophils in abdominal sepsis. Crit. Care Med..

[68] Kornerup K.N., Salmon G.P., Pitchford S.C., Liu W.L., Page C.P. (2010). Circulating Platelet-Neutrophil Complexes Are Important for Subsequent Neutrophil Activation and Migration. J. Appl. Physiol..

[69] Ziegler M., Wang X., Peter K. (2019). Platelets in cardiac ischaemia/reperfusion injury: A promising therapeutic target. Cardiovasc. Res..

[70] Makhoul S., Trabold K., Gambaryan S., Tenzer S., Pillitteri D., Walter U., Jurk K. (2019). cAMP- and cGMP-elevating agents inhibit GPIbalpha-mediated aggregation but not GPIbalpha-stimulated Syk activation in human platelets. Cell Commun. Signal..

[71] Fuentes F., Alarcón M., Badimon L., Fuentes M., Klotz K.N., Vilahur G., Kachler S., Padró T., Palomo I., Fuentes E. (2017). Guanosine exerts antiplatelet and antithrombotic properties through an adenosine-related cAMP-PKA signaling. Int. J. Cardiol..

[72] Klinger M., Freissmuth M., Nanoff C. (2002). Adenosine receptors: G protein-mediated signalling and the role of accessory proteins. Cell Signal..

[73] Fredholm B.B., Abbracchio M.P., Burnstock G., Daly J.W., Harden T.K., A Jacobson K., Leff P., Williams M. (1994). Nomenclature and classification of purinoceptors. Pharmacol. Rev..

[74] Ralevic V., Burnstock G. (1998). Receptors for purines and pyrimidines. Pharmacol. Rev..

[75] Wolska N., Rozalski M. (2019). Blood Platelet Adenosine Receptors as Potential Targets for Anti-Platelet Therapy. Int. J. Mol. Sci..

[76] Horn M., Bertling A., Brodde M.F., Müller A., Roth J., VAN Aken H., Jurk K., Heilmann C., Peters G., Kehrel B.E. (2012). Human neutrophil alpha-defensins induce formation of fibrinogen and thrombospondin-1 amyloid-like structures and activate platelets via glycoprotein IIb/IIIa. J. Thromb. Haemost..

[77] Zarbock A., Polanowska-Grabowska R.K., Ley K. (2007). Platelet-neutrophil-interactions: Linking hemostasis and inflammation. Blood Rev..

[78] Thomas M.R., Storey R.F. (2015). The role of platelets in inflammation. Thromb. Haemost..

[79] Rahman I., MacNee W. (1998). Role of transcription factors in inflammatory lung diseases. Thorax.

[80] Liverani E., Rico M.C., Tsygankov A.Y., Kilpatrick L.E., Kunapuli S.P. (2016). P2Y_12_ Receptor Modulates Sepsis-Induced Inflammation. Arter. Thromb. Vasc. Biol..

[81] Yan S.L., Russell J., Harris N.R., Senchenkova E.Y., Yildirim A., Granger D.N. (2013). Platelet abnormalities during colonic inflammation. Inflamm. Bowel Dis..

[82] Irving P.M., Macey M.G., Feakins R.M., Knowles C.H., Frye J.N., Liyanage S.H., Dorudi S., Williams N.S., Rampton D.S. (2008). Platelet-leucocyte aggregates form in the mesenteric vasculature in patients with ulcerative colitis. Eur. J. Gastroenterol. Hepatol..

[83] Schanze N., Bode C., Duerschmied D. (2019). Platelet Contributions to Myocardial Ischemia/Reperfusion Injury. Front. Immunol..

[84] Lisman T. (2018). Platelet-neutrophil interactions as drivers of inflammatory and thrombotic disease. Cell Tissue Res..

[85] Li J., Kim K., Barazia A., Tseng A., Cho J. (2015). Platelet-neutrophil interactions under thromboinflammatory conditions. Cell. Mol. Life Sci..

[86] Zuchtriegel G., Uhl B., Puhr-Westerheide D., Pörnbacher M., Lauber K., Krombach F., Reichel C.A. (2016). Platelets Guide Leukocytes to Their Sites of Extravasation. PLoS Biol..

[87] Evangelista V., Manarini S., Sideri R., Rotondo S., Martelli N., Piccoli A., Totani L., Piccardoni P., Vestweber D., De Gaetano G. (1999). Platelet/polymorphonuclear leukocyte interaction: P-selectin triggers protein-tyrosine phosphorylation-dependent CD11b/CD18 adhesion: Role of PSGL-1 as a signaling molecule. Blood.

[88] Theoret J.F., Uhl B., Puhr-Westerheide D., Pörnbacher M., Lauber K., Krombach F., Reichel C.A. (2001). P-selectin antagonism with recombinant p-selectin glycoprotein ligand-1 (rPSGL-Ig) inhibits circulating activated platelet binding to neutrophils induced by damaged arterial surfaces. J. Pharmacol. Exp. Ther..

[89] Gawaz M. (2004). Role of platelets in coronary thrombosis and reperfusion of ischemic myocardium. Cardiovasc. Res..

[90] Vickers J.D. (1999). Binding of polymerizing fibrin to integrin alpha(IIb)beta(3) on chymotrypsin-treated rabbit platelets decreases phosphatidylinositol 4,5-bisphosphate and increases cytoskeletal actin. Platelets.

[91] Vickers J.D., Kinlough-Rathbone R.L., Packham M.A., Mustard J.F. (1990). Changes in Phosphoinositides in Rabbit Platelets during Clot Formation. Comparison of Platelets Stimulated by ADP or by Thrombin in the Presence of Polymerising Fibrin. Platelets.

[92] Mehta J.L., Nicolini F.A., Donnelly W.H., Nichols W.W. (1992). Platelet-leukocyte-endothelial interactions in coronary artery disease. Am. J. Cardiol..

[93] Kuroda T., Shiohara E., Homma T., Furukawa Y., Chiba S. (1994). Effects of leukocyte and platelet depletion on ischemia--reperfusion injury to dog pancreas. Gastroenterology.

[94] Kuroda T., Shiohara E. (1996). Leukocyte and platelet depletion protects the liver from damage induced by cholestasis and ischemia-reperfusion in the dog. Scand. J. Gastroenterol..

[95] Guo Y., Tukaye D.N., Wu W.J., Zhu X., Book M., Tan W., Jones S.P., Rokosh G., Narumiya S., Li Q. (2012). The COX-2/PGI2 receptor axis plays an obligatory role in mediating the cardioprotection conferred by the late phase of ischemic preconditioning. PLoS ONE.

[96] Lee J., Ahn E., Park W.K., Park S. (2016). Phosphoproteome Profiling of SH-SY5y Neuroblastoma Cells Treated with Anesthetics: Sevoflurane and Isoflurane Affect the Phosphorylation of Proteins Involved in Cytoskeletal Regulation. PLoS ONE.

[97] Tsutsumi Y.M., Patel H.H., Lai N.C., Takahashi T., Head B.P., Roth D.M. (2006). Isoflurane produces sustained cardiac protection after ischemia-reperfusion injury in mice. Anesthesiology.

